# Circular RNA hsa_circ_0077837 is upregulated in non-small cell lung cancer to downregulate phosphatase and tensin homolog through methylation

**DOI:** 10.1080/21655979.2022.2025707

**Published:** 2022-03-04

**Authors:** Dezhi Li, Hongying Lv, Huijiang Gao, Zizong Wang, Dongfei Wang, Kaihua Tian, Lin Li

**Affiliations:** a Department of Respiratory and Critical Care Medicine, Shandong Provincial Hospital Affiliated to Shandong First Medical University, Jinan, P. R. China; b Oncology Department, The Affiliated Hospital of Qingdao University, Qingdao, P. R. China; c Thoracic Department, The Affiliated Hospital of Qingdao University, Qingdao, P. R. China; d Thoracic Department, Qingdao Municipal Hospital, Qingdao, P. R. China; e Department of Thoracic Surgery, Qilu Hospital of Shandong University, Jinan, P. R. China

**Keywords:** Non-small cell lung cancer, hsa_circ_0077837, PTEN, methylation, apoptosis

## Abstract

Circular RNA (circRNA) hsa_circ_0077837 inhibits colorectal cancer. Our research studied the participation of hsa_circ_0077837 in non-small cell lung cancer (NSCLC). Hsa_circ_0077837 and phosphatase and tensin homolog (PTEN) expression in cancer and paired non-cancer tissues from a total of 64 NSCLC patients were studied with RT-qPCR. To evaluate the prognostic value of hsa_circ_0077837 for NSCLC, these 64 patients were monitored for 5 years. Expression of PTEN in NSCLC cells with hsa_circ_0077837 overexpression was determined by RT-qPCR and Western blot. The methylation of PTEN gene in cells transfected with hsa_circ_0077837 expression vector was analyzed by methylation specific PCR (MSP). The roles of hsa_circ_0077837 and PTEN in NSCLC cell proliferation were evaluated using cell apoptosis assay. Our data showed that hsa_circ_0077837 was upregulated in NSCLC and predicted poor survival. Besides, hsa_circ_0077837 expression level was higher in 36 advanced cases (stage III and IV) than in 28 early-stage cases (stage I and II). Hsa_circ_0077837 was inversely correlated with PTEN across cancer tissues. In NSCLC cells, hsa_circ_0077837 overexpression decreased PTEN expression, increased PTEN gene methylation, and reduced HCC827 cell apoptosis via PTEN. Overall, hsa_circ_0077837 is upregulated in NSCLC and downregulates PTEN by increasing its gene methylation to suppress cell apoptosis.

List of abbreviations:

Non-small cell lung cancer (NSCLC); circRNAs (circular RNAs); methylation-specific PCR (MSP)

## Introduction

Lung cancer is the most common cancer in terms of incidence and mortality among males and females in most countries [1–3]. Compared to the 6% 5-year overall survival rate of small cell lung cancer patients (SCLC), more non-small cell lung cancer (NSCLC) patients (about 24%) can survive more than 5 years [4]. However, the overall survival is still poor and needs to be further improved. Although smoking contributes to NSCLC [5], a considerable number of NSCLC is also diagnosed in never-smokers [6], suggesting the complicated pathogenesis of NSCLC. Without timely and effective treatment, NSCLC may transform to SCLC, leading to even worse prognosis [7].

Although efforts have been made to prevent and treat NSCLC, patients’ survival has been significantly improved in recent decades [8]. Therefore, novel therapies are still needed. Studies have identified numerous molecular factors involved in the malignancy of NSCLC [9,10]. Although many of them could be targets for NSCLC treatment [11,12], more targets are needed to improve targeted therapy. Circular RNAs (CircRNAs) have no protein-coding capacity, but they regulate cancers by affecting protein production, suggesting their potentials as targets for targeted cancer therapy [13]. Moreover, circRNAs in lung cancer have been reported to be related to cancer progression, thereby affecting patients’ survival [13]. Some circRNAs with critical functions in NSCLC have been proven to be potential prognostic biomarkers [13]. CircRNA hsa_circ_0077837 inhibits colorectal cancer [14]. Our preliminary microarray data revealed that hsa_circ_0077837 is upregulated in NSCLC and inversely correlated with phosphatase and tensin homolog (PTEN), a critical tumor suppressor [15]. Therefore, it is reasonable to hypothesize that hsa_circ_0077837 might interact with PTEN to participate in NSCLC. Therefore, we investigated the potential interaction between hsa_circ_0077837 and PTEN in NSCLC.

## Methods

### Patients and tissue collection

From July 2013 to July 2015, 64 NSCLC patients (24 females and 40 males, 61.4 ± 9.8 years) were enrolled in Qilu Hospital of Shandong University after Ethics approval was obtained from the Ethics Committee. NSCLC was diagnosed by computed tomography (CT) scan and confirmed by histopathological biopsy. Patients were included if they were newly diagnosed and had not been treated previously. Patients were excluded if they were recurrent cases and complicated with other diseases. Primary tumors were resected from patients and dissected to isolate paired NSCLC and non-tumor tissues. All participants signed informed consent. The clinical characteristics of NSCLC patients are summarized in Table 1.

**Table 1.** Correlation between hsa_circ_0077837 expression and clinical characteristics in NSCLC patients (n = 64)

		hsa_circ_0077837		
Parameters	Number	Low (n = 32)	High (n = 32)	P value
Age				0.765
< 60	23	12	11	
≥ 60	21	20	21	
Gender				0.639
Male	20	19	21	
Female	24	13	11	
Tumor size (cm)				0.025*
< 3	24	15	9	
≥ 3	40	17	23	
Lymph-node metastasis				0.012*
Negative	22	16	6	
Positive	42	16	26	
TNM stage				0.013*
I–II	28	18	10	
III + IV	36	14	22	

*P < 0.05.

### Treatment and follow-up

The 64 NSCLC patients were grouped into the stage I or II (n = 28) and stage III or IV (n = 36) according to the 7th edition of the American Joint Committee on Cancer (AJCC) staging systems. All patients did not undergo treatments prior to the surgery. From the day of admission, patients were visited every month for 5 years to monitor their survival. The 64 patients either died of NSCLC during the follow-up or completed the follow-up.

### NSCLC cells and cell culture

NSCLC cell line HCC827 was obtained from ATCC and cultured in RPMI-1640 medium with 10% fetal bovine serum (FBS) to about 80% confluence for subsequent assays.

### Vectors and transfections

To overexpress hsa_circ_0077837 or PTEN, cells were transfected with hsa_circ_0077837 or PTEN pcDNA3.1 vector using Lipofectamine 2000 (Invitrogen). In each transfection, 1 µg hsa_circ_0077837 or PTEN expression vector was transfected into 10^8^ HCC827 cells.

### RNA preparations

Total RNAs were isolated from paired tissue samples and HCC827 cells using EZ RNA Miniprep Kit (EZ BioResearch) and treated with DNase I (Invitrogen) to remove genomic DNAs. RNA purity was checked by measuring OD 260/280 ratios.

### RT-qPCR

RNA samples were subject to reverse transcriptions to synthesize cDNA. hsa_circ_0077837 and PTEN mRNA levels were determined using RT-qPCRs with GAPDH as the internal control. The primer sets used for RT-qPCR were 5’-CCTGGAGAAACATGCCAAGGG-3’ and 5’-TCACTTCAGACACAGAGCCTACT-3’ for hsa_circ_0077837, 5’-GTTTACCGGCAGCATCAAA-3’ and 5’-CCCCCACTTTAGTGCACAG-3’ for PTEN, and 5’-AGCCTCCCGCTTCGCTCTC-3’ and 5’-GCGCCCAATACGACCAAATCCG-3’ for GAPDH. PCR data were processed using the 2^−ΔΔCT^ method.

### Immunohistochemical assay

Following formalin fixation and dehydration in ethanol, tissues were embedded in paraffin and prepared as 5 µm thick sections. The sections were blocked in 5% normal goat serum and were incubated in turn with rabbit anti-PTEN polyclonal antibody (1:800; ab31392, Abcam, Cambridge, UK) and goat anti-rabbit IgG (Alexa Fluor 594, Invitrogen). The sections were observed under a phase-contrast light microscope (Olympus) and photographed. Integrated optical density (IOD) was calculated using Image-Pro Plus software.

### Methylation-specific PCR (MSP)

A total of 5 μg isolated genomic DNAs were used for bisulfite modification using DNA Methylation-Gold^TM^ kit (ZYMO RESEARCH). After that, genomic DNA samples were used as templates to perform both routine PCRs and MSP using 5’-TAGATAGGTGCCCTTTGGGCCCTTG-3’ and 5’-CCCCCAAATCTGTGTCCTCATGGTGT-3’ for routine PCR and 5’-TAGATAGGTGTTTTTTGGGTTTTTG-3’ and 5’-CCCCCAAATCTATATCCTCATAATAT-3’ for MSP.

### Western blot analysis

Total proteins were isolated using RIPA solution (Invitrogen) and quantified using BCA assay. After denaturation, proteins were separated by 10% SDS-PAGE gel electrophoresis and transferred onto PVDF membranes. After blocking, the membranes were incubated in turn with GAPDH (ab8245, Abcam) or PTEN (ab31392, Abcam) primary antibodies and HRP IgG secondary antibody (ab6721, Abcam). Signals were then developed using ECL Western blotting Substrate Kit (ab65623, Abcam). Data were normalized using QuantityOne software.

### Cell apoptosis assay

HCC827 cells were cultured in a 96-well plate with 3,000 cells per well. Three wells were set for each experiment. After culturing for 48 h, cells were harvested, washed, and stained with Annexin-V FITC and PI. Finally, apoptotic cells were analyzed using FACSCalibur instrument.

### Subcellular fractionation analysis

Both nucleus and cytoplasm fractions of HCC827 cells were prepared using a Cell Fractionation Kit from Abcam (ab109719). In brief, cells were washed using ice-cold PBS and counted. A total of 10^7^cells were incubated with cell lysis buffer on ice for at least 20 min. The cell lysates were centrifuged for 10 min at 1200 g. The supernatants, which were the cytoplasm fractions, were collected, transferred to a new tube, and subjected to RNA isolation. Cell pellets, which were the nucleus fractions, were further incubated with nucleus lysis buffer for 10 min on ice and subjected to RNA isolation. Both RNA samples were prepared as cDNA samples and used to determine the expression of hsa_circ_0077837 using PCRs. GAPDH was included in this assay as a cytoplasm marker.

### Statistical analyses

Data were expressed as mean ± standard deviation (SD). Differences among multiple groups were analyzed using ANOVA Tukey’s test. The 64 patients were divided into high and low hsa_circ_0077837 level groups (n = 32, cutoff value = median hsa_circ_0077837 expression level in cancer tissue). Survival curves were plotted based on follow-up analysis and compared using log-rank test. The associations between patients’ clinical characteristics and hsa_circ_0077837 expression were analyzed using Chi-squared test. Correlations were analyzed by Pearson’s correlation coefficient. *P* < 0.05 was statistically significant.

## Results

### Altered hsa_circ_0077837 and PTEN expression in NSCLC

**Figure 1.**
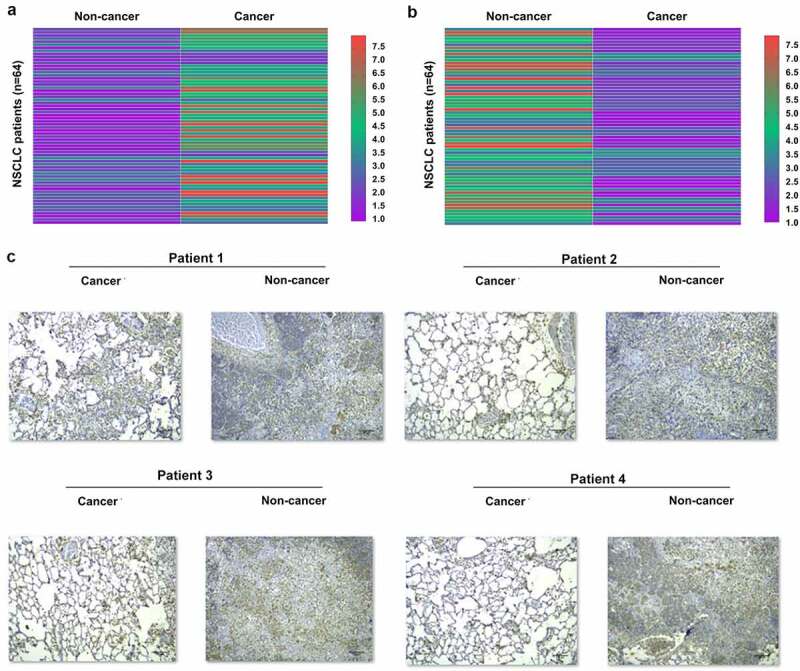
Hsa_circ_0077837 and PTEN expression in NSCLC. Hsa_circ_0077837 and PTEN mRNA expression levels in paired cancer and non-cancer tissue samples from 64 NSCLC patients were measured using RT-qPCR and used to plot heatmaps using Heml 1.0 software to reflect differential hsa_circ_0077837 expression in different TNM stages (data not shown). Immunohistochemical analysis was performed on paired tissues from four NSCLC patients to further analyze the differential PTEN expression in NSCLC (c). **, *p* < 0.01.

The expression levels of genes may indicate their functions. To this end, Hsa_circ_0077837 and PTEN expression levels in cancer and paired non-cancer tissue samples from 64 NSCLC patients were detected by RT-qPCR. The expression data of hsa_circ_0077837 and PTEN in paired tissues were used to plot heatmaps using Heml 1.0 software. The results showed that hsa_circ_0077837 was upregulated (Figure 1(a)), and PTEN was downregulated (Figure 1(b)) in cancer tissues. Besides, in these 64 cases of NSCLC, hsa_circ_0077837 expression levels were higher in the 36 advanced cases (stage III and IV) than in the 28 early-stage cases (stage I and II) (data not shown). Immunohistochemical analysis performed on paired tissues from four NSCLC patients showed that PTEN was downregulated in NSCLC tissues because the IOD value of cancer tissues was below 30% of the IOD value of non-cancer tissues in all cases (Figure 1(c)). Therefore, altered hsa_circ_0077837 and PTEN expression may participate in NSCLC.

### Correlations between hsa_circ_0077837 and PTEN

**Figure 2.**
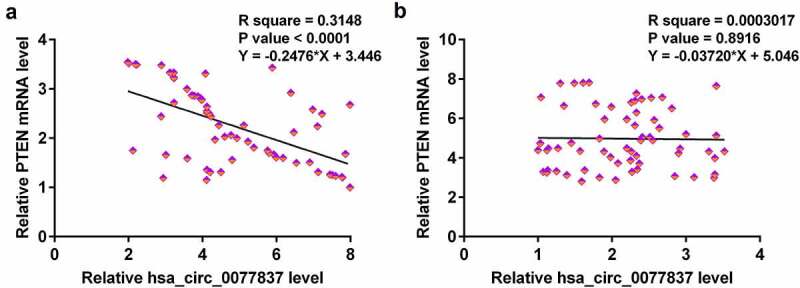
Hsa_circ_0077837 and PTEN levels were inversely correlated across cancer tissues. Correlations between hsa_circ_0077837 and PTEN mRNA across both cancer (a) and non-cancer (b) tissues were analyzed by Pearson’s correlation coefficient.

Correlations suggest interactions. Therefore, correlations between hsa_circ_0077837 and PTEN were explored with Pearson’s correlation coefficient. Hsa_circ_0077837 and PTEN were inversely correlated across cancer tissues (Figure 2(a)) but not across non-cancer tissues (Figure 2(b)). Therefore, hsa_circ_0077837 may interact with PTEN in NSCLC.

### Predictive value of hsa_circ_0077837 for patients’ survival

**Figure 3.**
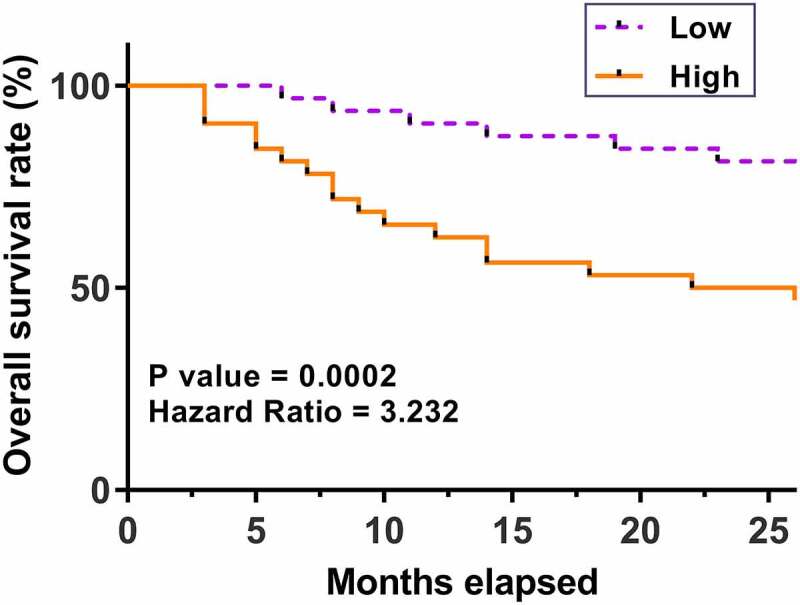
Predictive role of hsa_circ_0077837 for patients’ survival. The survival curves of patients with high and low hsa_circ_0077837 levels were plotted using data from follow-up study and compared using log-rank test.

The prognostic value of hsa_circ_0077837 for NSCLC was explored by plotting survival curves. Compared to patients with low hsa_circ_0077837 levels, patients with high hsa_circ_0077837 levels experienced worse survival (Figure 3). Therefore, high expression levels of hsa_circ_0077837 in cancer tissues may predict poor survival of NSCLC patients.

### Hsa_circ_0077837 overexpression decreased PTEN expression through methylation

**Figure 4.**
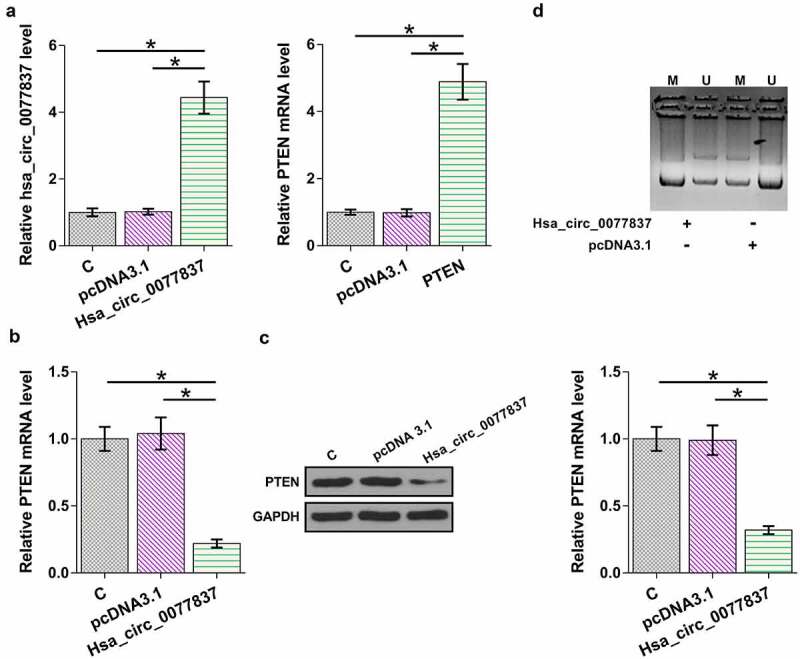
Hsa_circ_0077837 regulated PTEN methylation. HCC827 cells were overexpressed with hsa_circ_0077837 or PTEN (a). The effects of hsa_circ_0077837 overexpression on PTEN mRNA (b) and protein (c) levels were analyzed. The effect of hsa_circ_0077837 on PTEN gene methylation was analyzed by MSP (d). M, methylated PCR product; U, un-methylated PCR product. *, *p* < 0.05.

The inverse correlation between hsa_circ_0077837 and PTEN indicated their possible interaction. To further explore their relationship, HCC827 cells were overexpressed with hsa_circ_0077837 or PTEN. hsa_circ_0077837 or PTEN overexpression was confirmed by RT-qPCR (Figure 4(a), *p* < 0.05). Hsa_circ_0077837 overexpression decreased PTEN mRNA (Figure 4(b)) and protein (Figure 4(c)) expression (*p* < 0.05). The effect of hsa_circ_0077837 overexpression on PTEN gene methylation was evaluated by MSP. Compared to the empty vector group, cells transfected with hsa_circ_0077837 showed increased PTEN gene methylation (Figure 4(d)). Subcellular fractionation assay was used to determine the subcellular localization of hsa_circ_0077837. Hsa_circ_0077837 can be detected in both nucleus and cytoplasm fractions (Supplemental Fig.1), consistent with its function in regulating DNA methylation. Therefore, hsa_circ_0077837 may downregulate PTEN by increasing its gene methylation.

### Hsa_circ_0077837 overexpression reduced HCC827 cell apoptosis via PTEN

**Figure 5.**
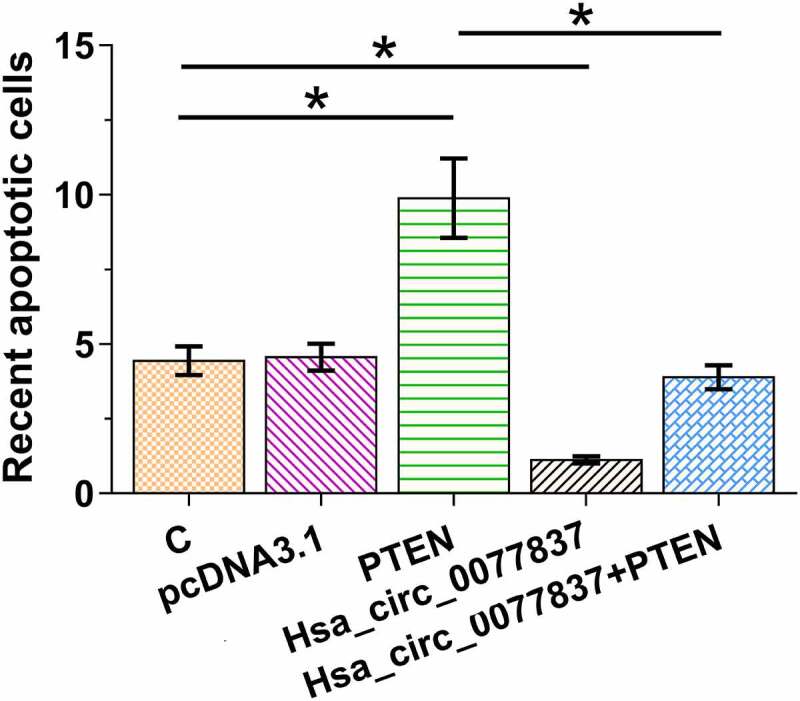
Hsa_circ_0077837 overexpression reduced HCC827 cell apoptosis via PTEN. The roles of hsa_circ_0077837 and PTEN in HCC827 cell apoptosis were explored using cell apoptosis assay. *, *p* < 0.05.

The role of hsa_circ_0077837 and PTEN in HCC827 cell apoptosis was analyzed. It was observed that hsa_circ_0077837 overexpression decreased HCC827 cell apoptosis, while PTEN overexpression increased HCC827 cell apoptosis. Moreover, PTEN overexpression reduced the inhibitory effect of hsa_circ_0077837 overexpression on cell apoptosis (Figure 5, *p* < 0.05). Therefore, hsa_circ_0077837 may suppress NSCLC cell apoptosis via PTEN.

## Discussion

This research studied the involvement of hsa_circ_0077837 in NSCLC. We found that hsa_circ_0077837 was upregulated in NSCLC and may downregulate PTEN by increasing PTEN gene methylation to suppress NSCLC cell apoptosis.

Hsa_circ_0077837 was under-expressed in colorectal cancer (CRC), and hsa_circ_0077837 overexpression suppressed CRC cell proliferation, suggesting the role of hsa_circ_0077837 as a tumor suppressor in CRC [14]. Our research revealed that hsa_circ_0077837 could suppress cell apoptosis, indicating it played an oncogenic role in NSCLC. Therefore, hsa_circ_0077837 may play different roles in different cancers, and the functions of hsa_circ_0077837 in other cancers should be investigated.

It has been well established that treatment strategies are closely related to the survival of patients [16]. Hsa_circ_0077837 expression was found to be closely correlated with the poor prognosis of NSCLC patients. Therefore, monitoring changes in hsa_circ_0077837 expression levels in NSCLC patients may improve the design of treatment approaches, thereby improving patients’ survival. However, the prognostic value of hsa_circ_0077837 for NSCLC remains to be further validated by more studies with a larger sample size.

PTEN is a tumor suppressor that participates in cancer biology by inducing cancer cell apoptosis via suppressing the PI3K-Akt pathway, a main cell survival pathway in cancers [15]. Consistently, our results showed downregulation of PTEN in NSCLC and its enhancing effect on NSCLC cell apoptosis. Recent advances in functional characterization of circRNAs have shown that circRNAs interact with methylation-related factors, such as TET1 and DNMT1, to regulate DNA methylation [17]. This study showed that hsa_circ_0077837 could increase PTEN gene methylation to downregulate its expression. Our findings enriched our understanding of the functionality of circRNAs. It is worth noting that hsa_circ_0077837 and PTEN levels were closely correlated across cancer tissues but not non-cancer tissues. Therefore, the interaction between hsa_circ_0077837 and PTEN may be mediated by certain pathological factors. The molecular mechanism that mediates their interaction remains to be further studied.

The roles of circRNAs in NSCLC have been extensively explored in recent years [18–20]. However, the functions of most circRNAs in NSCLC remain unclear and need to be investigated.

## Conclusions

Hsa_circ_0077837 is upregulated in NSCLC and predicts poor survival of NSCLC patients. Moreover, hsa_circ_0077837 may downregulate PTEN through methylation to suppress cancer cell apoptosis.

## Supplementary Material

Supplemental Material

## Data Availability

The datasets used and/or analyzed during the current study are available from the corresponding author on reasonable request.
