# Supplementary figures and images for: Circular RNA hsa_circ_0077837 is upregulated in non-small cell lung cancer to downregulate phosphatase and tensin homolog through methylation
Source: Bioengineered. 2022 Mar 4;13(3):6711–8. doi: 10.1080/21655979.2022.2025707 (PMC8973925; doi:10.1080/21655979.2022.2025707)

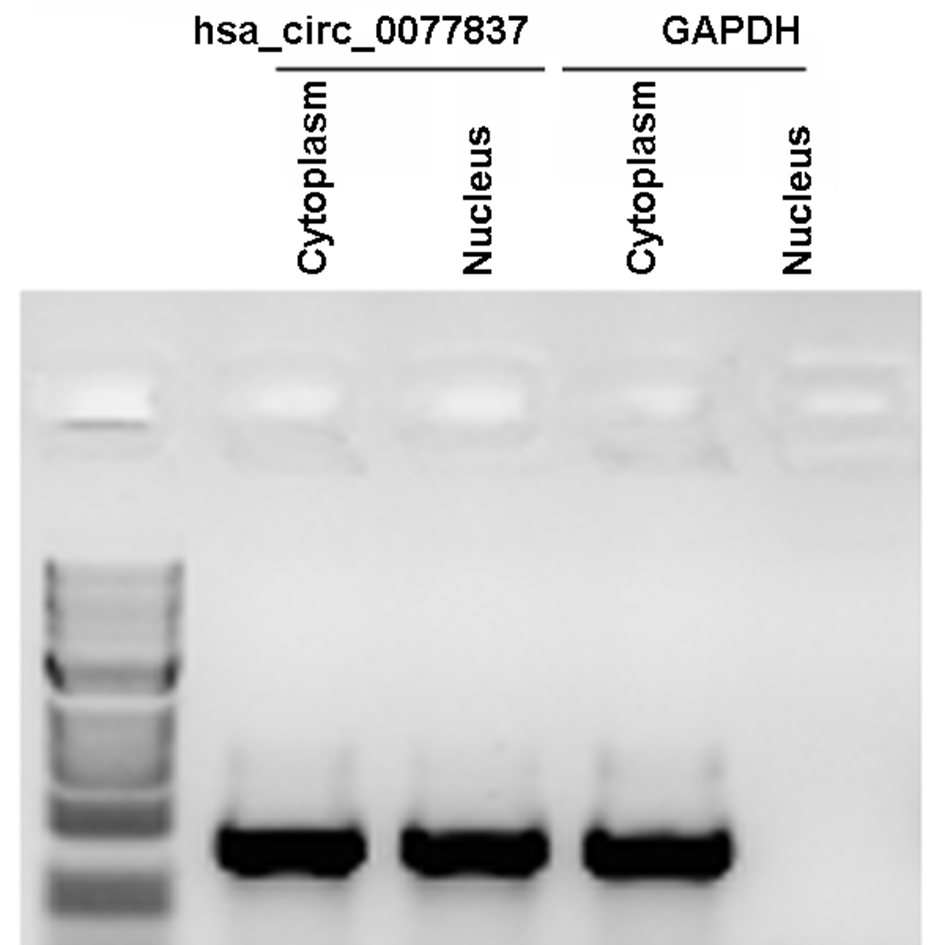

Supplement: Supplemental Material [file KBIE_A_2025707_SM4969.zip › supplementary/Supplemental Fig 1.tif]
